# Determinants of global cardiac implantable electrical device remote monitoring utilization – Results from an international survey

**DOI:** 10.1016/j.cvdhj.2024.03.003

**Published:** 2024-03-19

**Authors:** Bert Vandenberk, Neal Ferrick, Elaine Y. Wan, Sanjiv M. Narayan, Aileen M. Ferrick, Satish R. Raj

**Affiliations:** ∗ Department of Cardiology, University Hospitals Leuven, Leuven, Belgium; † Department of Cardiovascular Sciences, KU Leuven, Leuven, Belgium; ‡ Department of Cardiology, Montefiore Medical Center, New York, New York; § Division of Cardiology, Department of Medicine, College of Physicians and Surgeons, Columbia University, New York, New York; ‖ Cardiology Division, Cardiovascular Institute, Stanford University, Stanford, California; ¶ Cardiac Electrophysiology, White Plains Hospital, White Plains, New York, New York; # Department of Cardiac Sciences, Libin Cardiovascular Institute, Cumming School of Medicine, University of Calgary, Calgary, Canada; ∗∗ Autonomic Dysfunction Center, Division of Clinical Pharmacology, Department of Medicine, Vanderbilt University Medical Center, Nashville, Tennessee

**Keywords:** Remote monitoring, Cardiac implantable electronic devices, Device clinic, Survey, Compliance

## Abstract

**Background:**

Despite near-global availability of remote monitoring (RM) in patients with cardiac implantable electronic devices (CIED), there is a high geographical variability in the uptake and use of RM. The underlying reasons for this geographic disparity remain largely unknown.

**Objectives:**

To study the determinants of worldwide RM utilization and identify locoregional barriers of RM uptake.

**Methods:**

An international survey was administered to all CIED clinic personnel using the Heart Rhythm Society global network collecting demographic information, as well as information on the use of RM, the organization of the CIED clinic, and details on local reimbursement and clinic funding. The most complete response from each center was included in the current analysis. Stepwise forward multivariate linear regression was performed to identify determinants of the percentage of patients with a CIED on RM.

**Results:**

A total of 302 responses from 47 different countries were included, 61.3% by physicians and 62.3% from hospital-based CIED clinics. The median percentage of CIED patients on RM was 80% (interquartile range, 40–90). Predictors of RM use were gross national income per capita (0.76% per US$1000, 95% CI 0.72–1.00, *P* < .001), office-based clinics (7.48%, 95% CI 1.53–13.44, *P* = .014), and presence of clinic funding (per-patient payment model 7.90% [95% CI 0.63–15.17, *P* = .033); global budget 3.56% (95% CI -6.14 to 13.25, *P* = .471]).

**Conclusion:**

The high variability in RM utilization can partly be explained by economic and structural barriers that may warrant specific efforts by all stakeholders to increase RM utilization.

## Introduction

In patients with cardiac implantable electronic devices (CIED), remote monitoring (RM) has shown to improve survival, quality of life, and health care utilization.^1^^,^^2^ As such, RM has been recommended as part of the standard of care in patients with CIEDs.^2^^,^^3^ Despite the near-global availability of RM, there is a high geographical variability in the uptake and use of RM.^4^^,^^5^ For example, in the prospective ADVANCE cardiac resynchronization therapy (CRT) registry approximately 60% of patients enrolled in North and South America were on RM, while this was <6% in Asia.^5^ The underlying reasons for this geographic disparity remain largely unknown, but have been hypothesized to depend on reimbursement, personnel resources, and infrastructure.^1^^,^^2^ Therefore, the recent collaborative multiprofessional society expert consensus statement on RM called for novel data to identify local barriers that may limit the utilization of RM in order to develop strategies to optimize RM usage.^2^

An aim of this international survey included gathering global data on the use of RM and reimbursement to study the determinants of worldwide RM utilization and identify locoregional barriers of RM uptake.

## Methods

### Study design and participants

The intended target population of the survey included all CIED clinic personnel, including physicians, advanced practice providers, nurses, technicians, and others. An online survey was administered using the SurveyMonkey platform and distributed among the Heart Rhythm Society global network on December 8, 2022. All participants provided their individual informed consent electronically before proceeding with the survey. The questionnaire could be completed anonymously, but information on participants’ location and the name of their center were requested. All responses submitted by December 31, 2022, were analyzed. This study was a qualitative improvement initiative of the Heart Rhythm Society Digital Health Committee. The research reported in this paper adhered to the Helsinki Declaration as revised in 2013.

### Survey design

The survey was conducted in English and consisted of 40 questions in total. First, demographic information was collected, including the participant’s CIED clinic role, size of CIED clinic, and location (country, city, and name of center). Subsequently, information on the use of RM and organization of the CIED clinic was collected, including the percentage of patients enrolled in RM and frequency of remote CIED checks for each type of CIED. Lastly, information about local reimbursement and clinic funding was collected. The survey also included qualitative questions, which are beyond the scope of the current manuscript. In total, the survey required approximately 20 minutes to complete.

### Socioeconomic and geographical data

To study the impact of global socioeconomic and geographic differences on RM utilization, the following information was researched and collected for each country:

- Population density, expressed as people per square kilometer in 2021, as per data from the United Nations Statistics Division.^6^
- Gross national income (GNI) per capita, expressed in US dollars (World Bank Atlas method^7^). Countries were divided into high- and low-income countries, as per data from 2021 by the World Bank.^8^ The high-income threshold for 2021 was set at US$13,205 on July 1, 2022.^8^

### Statistical analysis

For this analysis, incomplete responses and responses by industry and third-party RM services were excluded. If multiple responses from the same center were submitted, the most complete response was included in this analysis. Continuous variables are presented as median and interquartile range (IQR), as all variables showed a non-normal distribution using the Kolmogorov-Smirnov test. Categorical variables are presented as numbers and percentages. Continuous parameters were compared between groups using the nonparametric Mann-Whitney *U* test or Kruskal-Wallis test. Categorical parameters were compared using χ^2^ tests. Univariable and multivariable linear regression was performed to identify determinants of the percentage of patients with a CIED on RM. Multivariable regression was performed using forward variable selection with a *P* value <.05 as entry criterion for addition to the model. The assumptions of linear regression analysis were verified visually using P-P plots and residuals vs fitted values plots. A *P* value <.05 was considered significant. Responses with missing answers were excluded from the analysis for those specific questions, so the reported percentages are adjusted for the number of available answers. Statistical analyses were performed using Stata version 17 (StataCorp LLC, College Station, TX) and SPSS version 29 (IBM Statistics, Armonk, NY). The world map graphs were made using an online tool, MapChart.^9^

## Results

### Respondent characteristics

**Figure 1:**
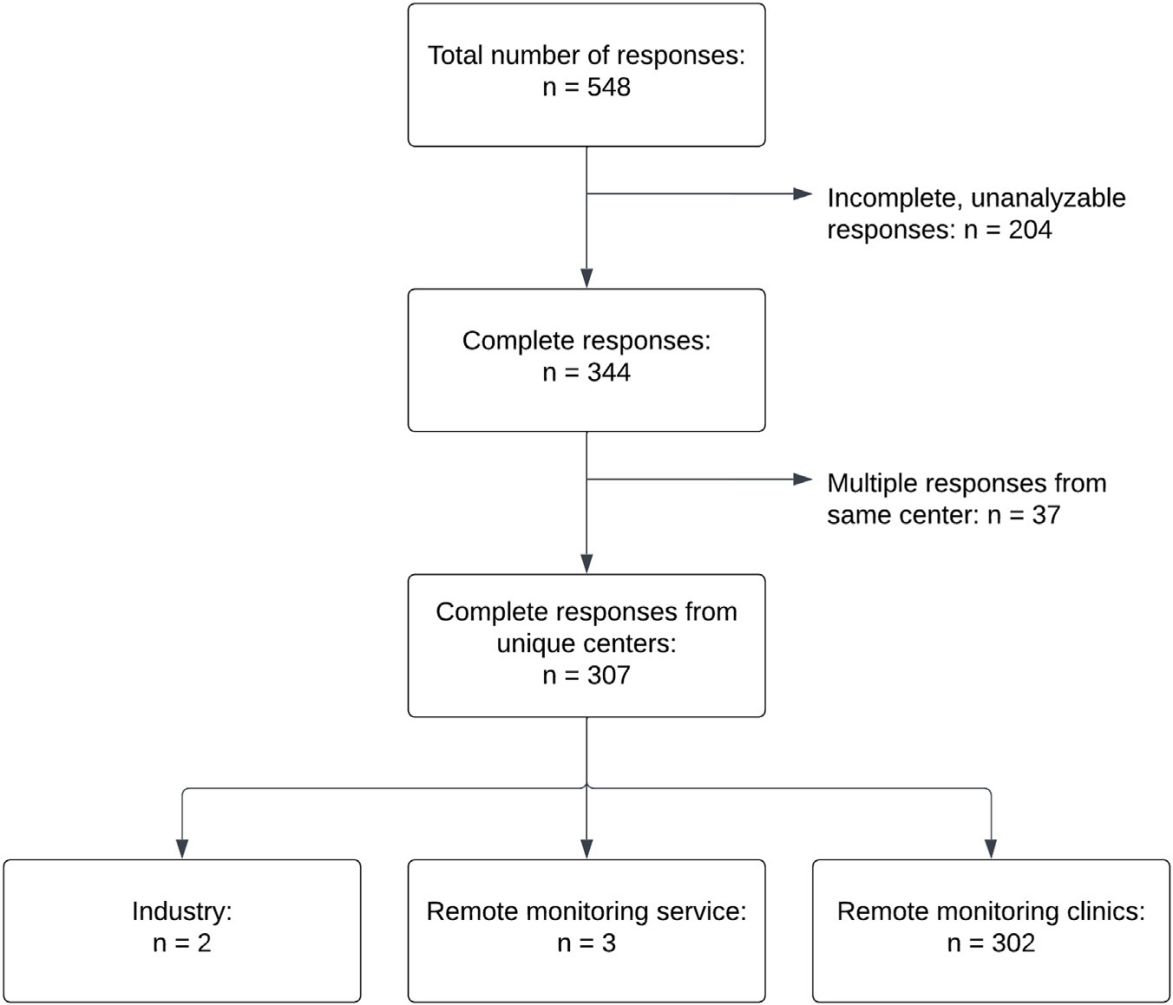
Data flow from the survey to the current analysis.

A total of 548 responses were received, of which 344 responses (62.8%) were complete (Figure 1). After excluding multiple responses from the same center (n = 37), as well as third-party services (n = 3) and responses from industry (n = 2), a total of 302 responses (55.1%) were included in this analysis.

**Figure 2:**
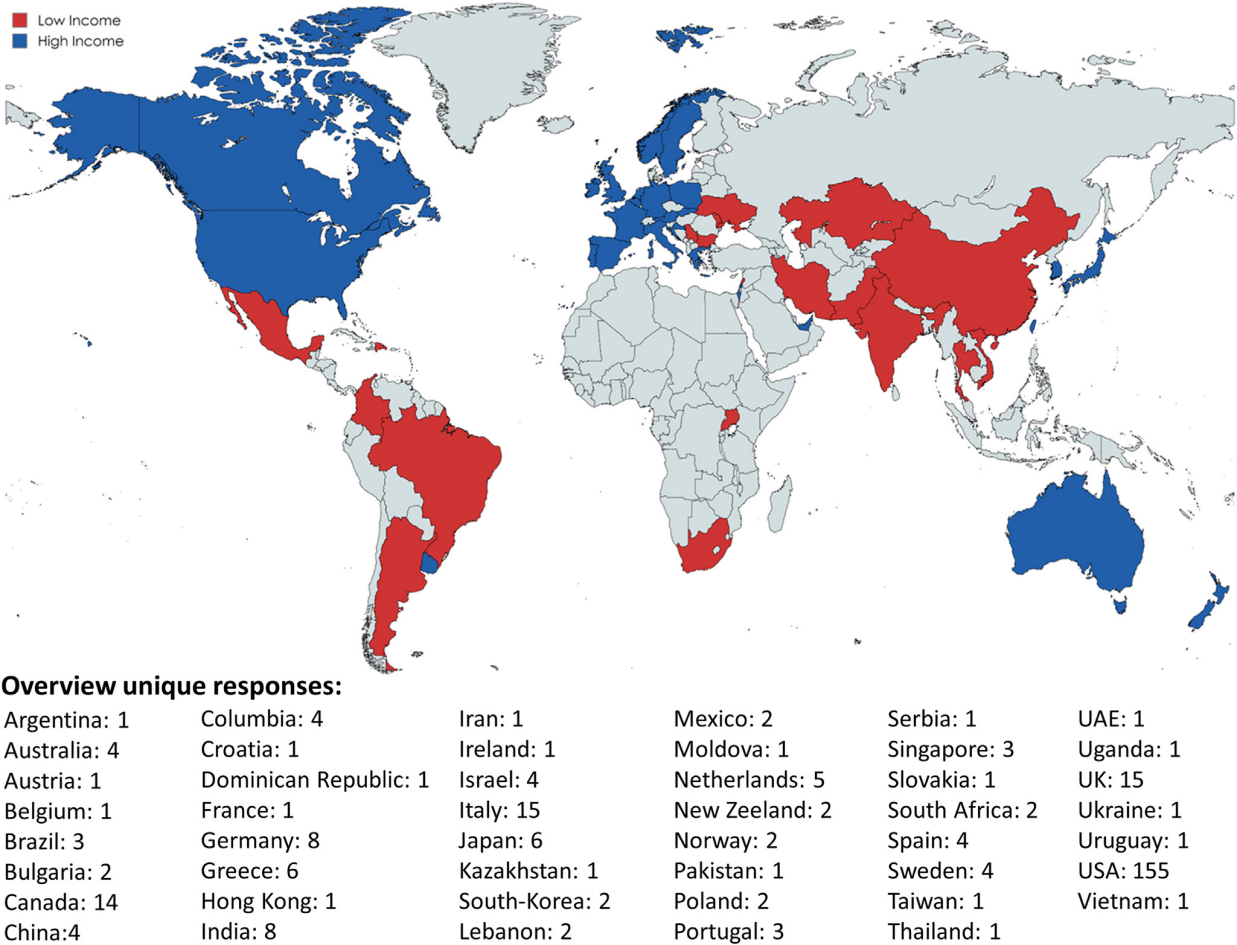
World map illustrating available responses with comparison of high- and low-income countries. Countries were categorized as high- and low-income based on the gross national income per capita. The high-income threshold for 2021 was set on July 1, 2022.^8^

The characteristics and origin of the available responses are presented in Figure 2 and Table 1. In total, responses from 47 different countries were received. Most responses were provided by physicians (61.3%), both adult and pediatric electrophysiologists, followed by nurses (16.2%) and nurse practitioners (7.3%). The majority of responses were from a hospital-based CIED clinic (62.3%) that operates only during business hours (76.1%). The median number of implanters per center was 4 (IQR 3–6) and a median of 2000 patients (IQR 700–4000) were followed in the CIED clinics of respondents, of which 80% (IQR 40–90) were on RM. The median patient to staff ratio was 750 (IQR 466–1100) and 58 centers (19.7%) used third-party RM services.

**Table 1:** Characteristics of survey respondents (N = 302)

Characteristic	Result
CIED clinic role	
Adult electrophysiologist	172 (57.0%)
Pediatric electrophysiologist	13 (4.3%)
Nurse practitioner	22 (7.3%)
Nurse	49 (16.2%)
Physician assistant	9 (3.0%)
Other	37 (12.2%)
CIED clinic type	
Hospital-based	188 (62.3%)
Officed-based	114 (37.8%)
CIED clinic operation	
Business hours	217 (76.1%)
24/7	85 (23.9%)
Number of implanters in center	4 (3–6)
Number of CIED patients followed /y	2000 (700–4000)
Percentage of CIED patients on RM (%)	80 (40–90)
Staff-to-patient ratio	750 (466–1100)
Use of third-party RM service	58 (19.7%)

CIED = cardiac implantable electronic device; RM = remote monitoring.

Continuous variables are presented as median and interquartile range (Q1–Q3). Categorical variables are presented as numbers and percentages.

### Determinants of global RM utilization

The results of univariable and multivariable linear regression analysis for determinants of the percentage of patients with CIEDs on RM are presented in Table 2. The GNI per capita, clinic type, and clinic funding were independently associated with the percentage of patients with CIEDs on RM. For each US$1000 GNI per capita, the percentage of patients on RM was on average 0.86% higher (95% CI 0.72%–1.00%). For example, the 2021 US GNI per capita was US$70,930, while this was US$8220 for the Dominican Republic. Office-based clinics were associated with a 7.48% higher percentage of patients on RM (95% CI 1.53%–13.44%). Lastly, when compared to CIED clinics without any clinic funding, clinics funded by a per-patient payment model were associated with a 7.90% (95% CI 0.63%–15.17%) higher percentage of patients on RM while clinics funded by a global budget model were associated with a nonsignificant 3.56% (95% CI -6.14% to 13.25%) higher percentage of patients on RM.

**Table 2:** Multivariable linear regression for percentage of patients with cardiac implantable electronic devices on remote monitoring

Variable	Univariable			Multivariable†		
	Beta	95% CI	*P* value	Beta	95% CI	*P* value
Population density (/ 100 inh/km^2^)	-0.26	-0.51 to 0.01	.051			
Gross national income per capita (/ US$1000)	0.99	0.87 to 1.11	<.001	0.86	0.72 to 1.00	<.001
Office-based clinic (vs hospital-based)	20.48	12.93 to 28.04	<.001	7.48	1.53 to 13.44	.014
Business hours (vs 24/7)	-2.88	-11.58 to 5.82	.515			
Number of implanting physicians	1.50	0.06 to 2.94	.041			
Total n of CIED patients followed annually (/ 100)	0.13	-0.01 to 0.27	.054			
Patient-to-staff ratio (/ 100)	0.16	-0.46 to 0.77	.619			
Clinic funding						
None	Ref.			Ref.		
Per-patient payment	31.48	23.48 to 39.49	<.001	7.90	0.63 to 15.17	.033
Global budget	13.80	1.68 to 25.91	.026	3.56	-6.14 to 13.25	.471

CIED = cardiac implantable electronic device; inh = inhabitants; km = kilometer; vs = versus.

R^2^ = 0.495; adjusted R^2^ = 0.487.

† Forward selection with entry if *P* value for addition to the model < .050. Constant of the final model = 4.03 (95% CI -5.59 to 13.65, *P* = .411).

### GNI per capita

For further analysis, countries were categorized into high- and low-income countries (Figure 2) based on the GNI per capita high income threshold for 2021, which was set at US$13,205.^8^ Table 3 provides a comparison of CIED clinics between high- and low-income countries. Responses from low-income countries were more often provided by physicians, most likely owing to the smaller CIED clinic team as the median patient-to-staff ratio was equal to the median number of CIED patients followed. CIED clinics in low-income countries were significantly smaller and had a very low utilization of RM when compared to CIED clinics in high-income countries (10.0% vs 85.0%, *P* < .001). CIED follow-up routine was not significantly different between high-income and low-income countries, but the funding for CIED clinic follow-up differed significantly, where 57.1% of CIED clinics in low-income countries did not have any funding while this was only 24.0% for CIED clinics in high-income countries (*P* < .001).

**Table 3:** Comparison of responses from high- and low-income countries

	High-income N = 264 (87.4%)	Low-income N = 38 (12.6%)	*P* value
Clinic role respondent – MD	152 (57.6%)	33 (86.8%)	<.001
Hospital-based CIED clinic	163 (61.7%)	28 (65.8%)	.630
CIED clinic operation – business hours	200 (78.1%)	17 (58.6%)	.020
Patient-to-staff ratio	760 (483–1150)	500 (300–1100)	.301
Number of implanters in center	4.0 (3.0–6.0)	4.0 (2.0–5.0)	.071
Number of CIED patients followed/y	2500 (1000–4725)	450 (165–1000)	<.001
<1000	63 (24.6%)	27 (71.1%)	<.001
1000–3000	105 (41.0%)	8 (21.1%)	
>3000	88 (34.4%)	3 (7.8%)	
Percentage of CIED patients on RM	85.0% (50.0%–91.0%)	10.0% (5.0%–30.0%)	<.001
Frequency of routine remote CIED checks (once per x months)			
Implantable loop recorders	1.0 (1.0–3.0)	2.5 (1.0–5.5)	.376
Pacemakers	3.0 (3.0–6.0)	5.5 (3.0–6.0)	.566
Implantable cardioverter-defibrillators	3.0 (3.0–6.0)	3.0 (3.0–6.0)	.949
Cardiac resynchronization therapy	3.0 (3.0–6.0)	3.0 (1.0–6.0)	.322
Heart failure diagnostics followed	155 (59.8%)	25 (71.4%)	.187
Frequency of HF diagnostics (once per x months)	3.0 (1.0–3.0)	3.0 (1.3–3.0)	.255
Percentage of remote checks that are alerts	20% (10%–40%)	15% (10%–37.5%)	.361
Remote CIED check time (min)	15.0 (6.1–23.8)	15.0 (10.0–30.0)	.391
Remote CIED check administration time (min)	15.0 (10.0–25.0)	15.0 (10.0–30.0)	.787
Remote reports provided to patients	131 (50.4%)	25 (69.4%)	.032
Clinic funding			
No funding	61 (24.0%)	20 (57.1%)	<.001
Per-patient payment	164 (64.6%)	11 (31.4%)	
Global budget	29 (11.4%)	4 (11.4%)	
Use of third-party RM service	54 (20.7%)	4 (11.8%)	.218

CIED = cardiac implantable electronic device; HF = heart failure; RM = remote monitoring.

Countries were categorized as high and low income based on the gross national income per capita. The high-income threshold for 2021 was set on July 1, 2022.^8^

Continuous variables are presented as median and interquartile range (Q1–Q3). Categorical variables are presented as numbers and percentages.

### Office-based vs hospital-based CIED clinics

A comparison by CIED clinic type is presented in Table 4. There was no difference in the median number of patients followed between office-based and hospital-based CIED clinics (2000 vs 2000; *P* = .0.662), but the percentage of patients on RM was significantly higher in office-based clinics (90.0% vs 70.0%, *P* < .001). The patient-to-staff ratio was nonsignificantly higher in office-based clinics (median 1000 vs 600; *P* = .057). The CIED follow-up routine was significantly more frequent for all CIED types and for heart failure diagnostics in office-based clinics, corresponding to a lower percentage of RM checks that are alerts in office-based clinics (17.5% vs 20.0%, *P* = .013). Most office-based clinics were funded by a per-patient payment model (75.2%), while this was only about half for the hospital-based clinics (51.7%, *P* < .001). Lastly, office-based clinics more often used third-party RM services (31.5% vs 12.5%; *P* < .001).

**Table 4:** Comparison of office-based and hospital-based cardiac implantable electronic device clinics

	Hospital-based N = 188 (62.3%)	Office-based N = 114 (37.3%)	*P* value
Clinic role respondent – MD	128 (68.1%)	57 (50.0%)	.002
CIED clinic operation – business hours	132 (74.2%)	85 (79.4%)	.311
Patient to staff ratio	600 (395–1000)	1000 (500–1250)	.057
High-income country	163 (86.7%)	101 (88.6%)	.630
Number of implanters	4 (3–6)	4 (2–5)	.169
Number of CIED patients followed/y	2000 (600–3500)	2000 (725–4230)	.662
<1000	56 (30.9%)	34 (30.1%)	.546
1000–3000	73 (40.3%)	40 (35.4%)	
>3000	52 (28.7%)	39 (34.5%)	
Percentage of CIED patients on RM	70.0% (28.8%–90.0%)	90.0% (75.6%–95.0%)	<.001
Frequency of routine remote CIED checks (once per x months)			
Implantable loop recorders	3 (1–6)	1 (1–3)	<.001
Pacemakers	4 (3–6)	3 (3–4)	.003
Implantable cardioverter-defibrillators	3 (3–6)	3 (3–3)	.027
Cardiac resynchronization therapy	3 (3–6)	3 (3–3)	.031
Heart failure diagnostics followed	103 (55.7%)	77 (70.6%)	.011
Frequency of HF diagnostics (once per x months)	3 (1–3)	1 (1–3)	<.001
Percentage of remote checks that are alerts	20.0% (10.0%–50.0%)	17.5% (10.0%–30.0%)	.013
Remote CIED check time (min)	15 (8.5–30.0)	15.0 (7.3–20.0)	.566
Remote CIED check administration time (min)	18 (10–25)	15 (10–22.5)	.437
Remote reports provided to patients	92 (49.5%)	64 (58.2%)	.147
Clinic funding			
No funding	64 (35.6%)	17 (15.6%)	<.001
Per-patient payment	93 (51.7%)	82 (75.2%)	
Global budget	23 (12.8%)	10 (9.2%)	
Use of third-party RM service	23 (12.5%)	35 (31.5%)	<.001

CIED = cardiac implantable electronic device; HF = heart failure; RM = remote monitoring.

Continuous variables are presented as median and interquartile range (Q1–Q3). Categorical variables are presented as numbers and percentages.

### Clinic funding

**Figure 3:**
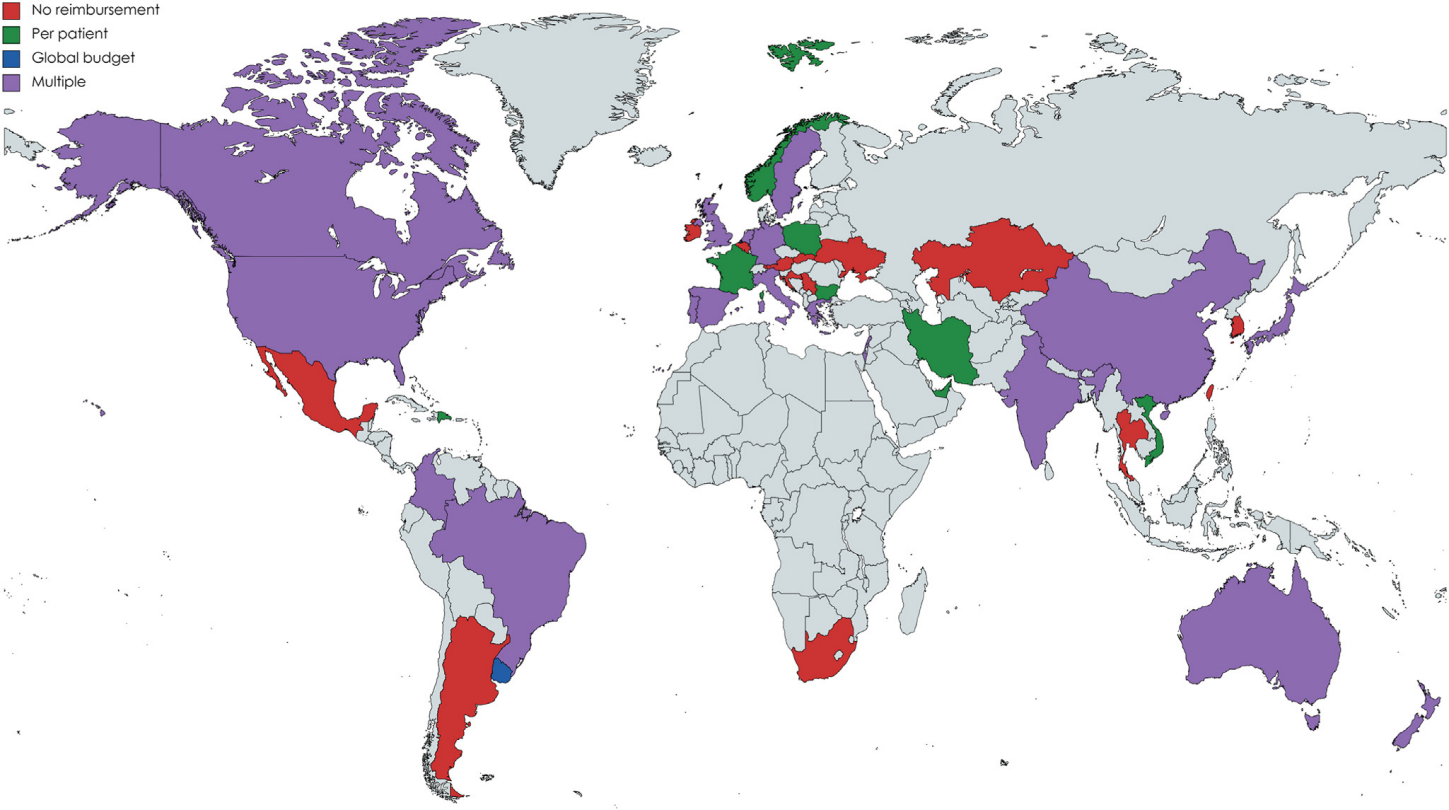
World map illustrating the global heterogeneity in cardiac implantable electronic device clinic funding models.

A comparison by clinic funding model is presented in Table 5. Overall, there was a large heterogeneity in CIED clinic funding models, as presented in Figure 3. In 19 countries, more than 1 different CIED clinic funding model was reported by the respondents. Besides the previously reported differences, the median number of CIED patients followed differed significantly between the different CIED clinic funding models (median: no funding 1200 vs per-patient payment 2000 vs global budget 3050; *P* = .010). The frequency of remote CIED checks and heart failure diagnostics was significantly higher in CIED clinics with a per-patient payment model when compared to no clinic funding and funding by global budget (Table 5). The use of third-party RM services was significantly higher in CIED clinics with per-patient payment models (no funding 5% vs per-patient payment 27.0% vs global budget 6.1%; *P* < .001).

**Table 5:** Comparison of clinic funding models

	No funding N = 81 (28.0%)	Per patient payment N = 175 (60.6%)	Global budget N = 33 (11.4%)	*P* value
Clinic role respondent – MD	63 (77.8%)	94 (53.7%)	23 (69.7%)	<.001
Hospital-based CIED clinic	64 (79.0%)	93 (53.1%)	23 (69.7%)	<.001
CIED clinic operation – business hours	53 (72.6%)	132 (76.6%)	25 (80.6%)	.643
Patient-to-staff ratio	800 (300–1000)	700 (500–1200)	850 (500–1500)	.778
High-income country	61 (75.3%)	164 (93.7%)	29 (87.9%)	<.001
Number of implanters	4 (3–5)	4 (3–6)	4 (3–7)	.568
Number of CIED patients followed/y	1200 (562.5–3000)	2000 (700–4203)	3050 (1600–5500)	.010
<1000	27 (33.8%)	54 (31.4%)	5 (15.2%)	.015
1000–3000	37 (46.3%)	62 (36.0%)	11 (33.3%)	
>3000	16 (20.0%)	56 (32.6%)	17 (51.5%)	
Percentage of CIED patients on RM	40% (10%–75%)	90% (70%–95%)	70% (25%–85%)	<.001
Frequency of routine remote CIED checks (once per x months)				
Implantable loop recorders	3 (1–6)	1 (1–3)	3 (1–6)	.002
Pacemakers	6 (3–6)	3 (3–6)	6 (3–6)	<.001
Implantable cardioverter-defibrillators	4 (3–6)	3 (3–3)	3 (3–6)	.056
Cardiac resynchronization therapy	3 (3–6)	3 (3–3)	3 (3–6)	.024
Heart failure diagnostics followed	55 (68.8%)	100 (57.1%)	20 (61.6%)	.212
Frequency of HF diagnostics (once per x months)	3 (1.3–3.8)	1 (1–3)	3 (3–3)	<.001
Percentage of remote checks that are alerts	22.5% (10%–50%)	20% (10%–38.8%)	20% (10%–42.5%)	.372
Remote CIED check time (min)	15 (8–30)	15 (7.5–20)	15 (5–20)	.675
Remote CIED check administration time (min)	15 (10–22.5)	15 (10–25)	15 (10–30)	.879
Remote reports provided to patients	40 (49.4%)	95 (54.6%)	17 (51.5%)	.731
Use of third party RM service	5 (6.2%)	47 (27.0%)	2 (6.1%)	<.001

CIED = cardiac implantable electronic device; HF = heart failure; RM = remote monitoring.

Continuous variables are presented as median and interquartile range (Q1–Q3). Categorical variables are presented as numbers and percentages.

## Discussion

This international survey on CIED clinic organization for RM in patients with CIEDs aimed to improve the understanding of global RM utilization in the post-COVID era. There was a high variability in the use of RM across the globe, which was associated with the CIED clinic type, the country’s GNI per capita, and the funding model of the CIED clinic.

While the number of CIED manufacturers is limited and all have a near-global availability, RM utilization is highly variable across the globe. Lau and colleagues^4^ reported a high variability in the use of RM in the Asia-Pacific region in 2013. While the RM utilization was very low in Southeast Asia, India, and Hong Kong, the RM utilization in Australia, New Zealand, and Japan was around 50% in patients with implantable cardioverter-defibrillator (ICD) and CRT devices.^4^ More recent data from 2022 by Varma and colleagues^5^ showed that this discrepancy between Asia and America persists, albeit they could not identify the underlying reason. The results of this survey reflect the post-COVID era. A recent European survey showed a significant increase in RM utilization for pacemakers and implantable loop recorders, while there was no difference for ICD and CRT devices.^10^ In this European survey, RM utilization was highest in patients with ICDs, at 69.6%, which is still lower compared to the observed median RM utilization in our survey. This emphasizes the geographical discrepancies discussed above and the need for more detailed insights in global trends in RM use. Further, even within countries the uptake of RM varies significantly.^11^ A socioeconomic analysis in the United States showed that lack of health insurance, unemployment, lower median income, and living in an urban neighborhood were associated with less RM utilization.^11^ To the best of our knowledge, this international survey is the first to collect global data on the use of RM and to identify determinants of RM utilization on a global basis.

In our survey, there was no significant difference in the frequency of routine remote CIED checks and heart failure diagnostics between high-income and low-income countries, but the frequency of remote CIED checks and heart failure diagnostics was significantly higher in office-based CIED clinics and in clinics with a per-patient payment funding model. Furthermore, third-party RM services were more frequently used by CIED clinics with a per-patient payment funding model. This may result from a financial incentive for CIED clinics where RM, besides providing the recommended clinical service, may be used as a business model. Conversely, the lack of such a financial incentive might prevent the provision of this recommended clinical service. This economic and financial barrier has been identified before. In 2015, a European survey identified the lack of reimbursement as the most important barrier for RM implementation in Europe, followed by the increased workload and lack of infrastructure.^2^^,^^12^ Similar findings were reported by a serial analysis of 2 surveys in Italy over a 5-year period, where 86.7% of centers reported that the lack of reimbursement was the main barrier to implement RM.^13^

Identifying the socioeconomic and structural barriers raises the question of how to improve care for these patients. Given the proven benefits of RM and that it is considered standard of care, the overall aim would be to increase RM utilization at a global level.^2^^,^^14^ This includes providing the CIED patients in low-income countries with the same benefits associated with RM as CIED patients in high-income countries, but also decreasing these disparities within a single country. This will require identifying local barriers and then specific efforts by health care providers, CIED manufacturers, third-party RM services, and patients to overcome these barriers and improve RM utilization.^2^ Future research should aim to identify specific hurdles that can be addressed, such as mobile network and Wi-Fi access, but also nonfinancial determinants such as details on culture and education programs. RM utilization (provision of RM to the patient) is insufficient, and attention must be paid to increasing RM adherence (RM continuous connectivity by the patient) as well.^2^^,^^11^^,^^15^

### Limitations

The results from this survey should be interpreted along with the inherent limitations. First, as the survey was distributed by the Heart Rhythm Society Digital Health Committee, the number of invitations sent will be several orders of magnitude larger than the number of responses received, resulting in a risk for selection bias that may affect the results. Second, more than half of the included responses were from the United States and several other countries had a low number of responses. A sensitivity analysis was performed in which the number of responses from the United States was reduced by random sampling down to a third of available responses, and the results of the multivariate linear regression remained significant. Third, no data on market shares for CIED vendors were available, nor did the survey address whether there was a price difference for RM-enabled devices or services for the patient. Further, data on mobile network access and access to Wi-Fi were not available. Fourth, reported numbers and details on the CIED clinic may represent rough estimates; however, in surveys one must assume that respondents report data to the best of their knowledge. Finally, several nonfinancial variables of interest were not collected in the survey, for example cultural differences and details on literacy and education programs.

## Conclusion

Worldwide, there is a high variability in RM utilization, which was associated with the gross national income per capita, office- vs hospital-based clinics, and the CIED clinic funding model. The frequency of routine RM CIED checks and heart failure diagnostics was significantly higher in CIED clinics funded by per-patient payment models, suggesting that financial incentive can increase RM utilization. Identification of these economic and structural barriers should be followed by implementation of appropriate reimbursement models adapted to the local health care systems. This could not only increase RM utilization, but also optimize CIED clinic workflows to cope with the associated data load when increasing RM uptake.
